# Policing sexuality: Sexual minority youth, police contact, and health inequity

**DOI:** 10.1016/j.ssmph.2022.101292

**Published:** 2022-11-17

**Authors:** Gabriel L. Schwartz, Jaquelyn L. Jahn, Amanda Geller

**Affiliations:** a UCSF Philip R. Lee Institute for Health Policy Studies, 490 Illinois St, Floor 7, San Francisco, CA, 94158, USA; b The Ubuntu Center on Racism, Global Movements, & Population Health Equity, Drexel University Dornsife School of Public Health, 3600 Market St, Philadelphia, PA, 19104, USA; c University of California, Irvine, Department of Criminology, Law and Society, 2340 Social Ecology II, Irvine, CA, 92697-7080, USA

**Keywords:** Sexual minorities, LGBTQ health, Health inequities, Social determinants of health, Policing, Criminal legal system, Youth

## Abstract

Police contact is increasingly recognized as an adverse childhood experience and determinant of poor mental health. While targeting of LGBTQ sex and community spaces by law enforcement has a long precedent in US history—and while LGBTQ people continue to protest unfair police treatment—little population-level research has examined police contact disparities by sexual orientation or gender identity. We test whether sexual minority (SM) youth have higher risk of police contact through young adulthood. We analyze a nationally representative cohort of >15,000 US young adults who were in middle/high school in the mid-1990s, with police contact histories collected at age 18–25. Using four different, equally reasonable approaches to coding youth-reported sexual orientation, we identified ∼500–1900 SMs. Compared to heterosexual youth, SM youth had 1.86 times the odds of ever being stopped by police (95% CI = 1.56–2.22, p < 0.001), were stopped 1.60 times as often (CI = 1.38–1.86, p < 0.001), and were stopped at younger ages (survival time ratio = 0.91, CI = 0.88–0.93, p < 0.001). Inequities were particularly driven by SM women, among whom disparities were severe (ever stopped OR = 2.18, stop count ratio = 2.44, survival time ratio = 0.87). For men, inequities only emerged once a broad definition of SM was adopted, suggesting that young SM men who do not identify as LGB (or who are reticent to report themselves as such) may be at particular risk. Results were robust to adjustment for race/ethnicity and parental nativity, though small cells meant models stratified by race/ethnicity were underpowered.

Given substantially heightened police contact among SM youth (particularly, young SM women), care providers and educators working with them should explicitly combat homophobic and criminal legal system stigma and screen for police contact and its psychological sequelae. More data on LGBTQ communities’ criminal legal system contact throughout life is essential for preventing the causes and consequences of related sexual orientation-based health inequities.

## Highlights

• Police contact is an adverse childhood experience that impacts health.
• We examine police contact inequities across sexual minority (SM) status among youth.
• Police more likely to stop SMs, stop SMs more times, & stop SMs at younger ages.
• Inequities were more severe among young SM women than young SM men.
• Police contact inequities may cause & exacerbate sexuality-based health inequities.

## 1 Introduction

Exposure to the police is common among contemporary American youth (Geller, 2021) and is increasingly recognized as a risk factor for poor health, especially poor mental health (Geller et al., 2014; Krieger et al., 2015; Sewell & Jefferson, 2016). Its distribution in the population is thus of crucial public health importance: inequitably distributed causes of poor health generate health inequities. Researchers are beginning to flesh out the ways structural racism manifests in police stop patterns, showing that Black and Hispanic youth experience greater and/or more intrusive police contact than their White peers (Geller, 2021). But less is known about police contact across other demographic markers of exposure to structural oppression, including sexual orientation (a marker of exposure to homophobia/heterosexism, including social and economic discrimination, stigma, and violence) (Bernstein et al., 2003; Hatzenbuehler et al., 2013). Describing sexual minorities' experiences with early-stage criminal legal system involvement is critical given that lesbian, gay and bisexual youth are overrepresented in carceral facilities (Wilson et al., 2017) and articulate interactions with police as a source of “terror,” (Levin, 2019) placing their well-being at risk (Wildeman & Wang, 2017). In an era of political movements for the equal treatment and well-being of sexual minorities (SMs) and of communities affected by mass incarceration, policymakers and public health practitioners require accurate data on SMs’ policing exposures throughout life.

Multiple mechanisms suggest police contact as a social determinant of poor health (Geller et al., 2014). Most directly, the invasive conduct of many police stops (Geller, 2021) creates physical injury risks (Edwards et al., 2019; Feldman et al., 2016). The acute stress of an incident may also undermine health (Mendelson et al., 2008), especially if it co-occurs with chronic stress tied to the low social position of many individuals stopped by the police (Sapolsky, 2004). Further, an aggressive encounter may trigger anticipatory stress if the person stopped subsequently lives in a state of fear and heightened vigilance that similar incidents might happen again (Geller et al., 2014). Finally, the psychosocial meaning of police stops, particularly for those who attract suspicion despite doing nothing illegal, may carry a stigma that triggers depressive symptoms. Individuals who perceive that they were unfairly stopped due to their identity—e.g., race, ethnicity, or sexual orientation—may be particularly vulnerable (Anderson, 2013; Everson-Rose et al., 2015; Krieger, 2015; Sawyer et al., 2012).

This has implications for LGBTQ health, given the long history of mistreatment of SMs by American law enforcement (Mallory et al., 2015). Homosexual sex was itself criminalized in many states until 2003, and police have historically targeted establishments patronized by SMs (e.g., gay bars) for aggressive enforcement of liquor licensing laws, laws against public lewdness, and other discretionary enforcement (Mallory et al., 2015). In contemporary surveys, self-identified lesbian, gay, bisexual, or questioning youth report more negative police interactions than those identifying as heterosexual (Stoudt et al., 2012). And in interviews from large cities such as New York, transgender people (the majority of whom identify as sexual minorities (James et al., 2016), and whose gender presentation may be interpreted by cis-heterosexual people as reflecting sexual minority status (Spielmann & Stern, 2021)) report particularly severe harassment, including spurious accusations of prostitution, arrests justified by their possession of condoms, and sexual assaults by police officers (Make The Road New York, 2012).

Such experiences have the potential to exacerbate existing health inequities burdening SMs—in stress, mental health, violence victimization, substance abuse, sexually transmitted infections, and cardiovascular disease, among other outcomes (Caceres et al., 2020; Connors et al., 2020)—and contribute to their experiences of homophobic stigma (Meyer & Northridge, 2007). Aggressive police encounters may serve as manifestations of structural stigma (Link & Phelan, 2001), which threatens SMs’ health (Hatzenbuehler et al., 2009, 2010) and drives health inequities (Hatzenbuehler et al., 2013). For example, men who self-reported having sex with men (MSM) and reported police harassment also reported more severe internalized homophobia and depressive symptoms than their un-harassed MSM peers (Khan et al., 2018). The use of homophobic invective by police (Brunson & Weitzer, 2009) may compound these responses. Disproportionate policing could also be a driver of stark inequities in incarceration rates between heterosexual and SM people in both youth and adulthood (Meyer et al., 2017; Movement Advancement Project, 2017), with long-term implications for inequities in social, economic, and physical well-being (American Public Health Association, 2020; Wildeman & Wang, 2017).

Police contact is a public health concern for all SMs, but particularly for developing youth. Adolescence is a period of rapid physical, psycho-cognitive, and social change (Choudhury et al., 2006; Fuhrmann et al., 2015; Romeo, 2013). Adolescent experiences, positive and negative, often have lasting consequences for health and wellbeing. Adolescence is also the period of sexual maturity, and often the life stage in which many people first identify as a SM and begin experiencing homophobic encounters that explicitly target them. For the process of sexual maturation to also be a process of discriminatory minoritization may have lifelong psychological and health consequences.

Despite the potential importance of police encounters as a social determinant of SM health, we know little about the policing experiences of SMs on a population level. This paper uses population-based data to quantify the police exposure of SM youth and measure the extent to which they might experience more contact than their heterosexual peers.

## 2 Methods

### 2.1 Data

We analyzed data from the National Longitudinal Study of Adolescent to Adult Health (Add Health), a nationally representative cohort of 20,745 7th through 12th graders recruited during the 1994–1995 school year (Wave 1) and followed into adulthood. Among the study's follow-up waves was a survey collected in 2000–2001 (Wave 3), when respondents were 18–26. Wave 3 asked its 15,197 respondents questions both about their sexual orientation and their experiences with police, providing one of the only large, randomly selected, population-based datasets in the US that includes sexual orientation and criminal legal system data together.

### 2.2 Defining sexual minority status

A variety of potential definitions for “sexual minority” are reasonable; we thus use four different definitions that were derive-able using Add Health data and provide estimates for each. These definitions are based on three questions. The first asked participants to “choose the description that best fits how [they thought] about [themselves],” providing the response options “100% heterosexual (straight)”, “mostly heterosexual (straight), but somewhat attracted to people of [their] own sex”, “bisexual – that is, attracted to men and women equally”, “mostly homosexual (gay), but somewhat attracted to people of the opposite sex”, “100% homosexual (gay)”, “not sexually attracted to either males or females”, or “don't know.” The second and third questions asked whether respondents had ever had a romantic attraction to a “male” or “female” person, respectively. We then combined these questions with Add Health's self-reported data on binary sex to determine whether a given respondent had reported same-sex attraction—noting that Add Health did not collect data on gender identity or expression, which limited our ability to fully classify LGBTQ participants, particularly those who are transgender or non-binary.

From these questions, we created four definitions:

1. Identified as lesbian, gay, or bisexual: responded “bisexual,” “mostly homosexual,” or “100% homosexual”
2. As in Definition 1, but also including those who reported ever being romantically attracted to someone of their same binary sex
3. As in Definition 2, but also including those who responded “mostly heterosexual (straight), but somewhat attracted to people of [their] own sex”
4. As in Definition 3, but also including those who reported that they did not know how to describe themselves

Unlike in SM Definition 4, those who said they did not know how to describe their sexual orientation were considered missing in Definitions 1, 2, and 3. People who were not sexually attracted to “either males or females” were coded as asexual and included as their own category with a separate dummy variable. Due to untenably low numbers of asexual respondents (extremely low statistical power), we present estimates only for non-asexual sexual minorities.

Add Health did not include a question about who participants had had sex with at the time of Wave 3, so definitions based on sexual activity were not available.

### 2.3 Police contact outcomes

Add Health respondents were asked whether they had ever been stopped by the police (binary), the number of times they had been stopped by the police (count), and the age when they were first stopped by the police. We treat these as three separate outcomes.

### 2.4 Covariates

Our analysis proceeds from the understanding that being attracted to people of a given sex or gender is effectively driven by chance, or by social structures that affect everyone within a society (Akpan, 2019; Ganna et al., 2019; Långström et al., 2010). That is, few individual-level traits *cause* same-sex attraction, so only a few variables could be confounders (for a directed acyclic graph, see Appendix 1). Here, we include age (given that police contact risk increases, and sexual identity develops, as children age) and gender. Gender is included because it determines exposures to gender oppression. This gender oppression differentially shapes expressions of queer sexual desire for men, women, and non-binary people (Fields et al., 2015; Richards, 2015), as well as differentially targets men, women, and non-binary people for police surveillance and incarceration (Geller, 2021; Wagner, 2012), creating confounding and effect heterogeneity. We proxy for gender oppression exposures with binary sex (with female participants, proxying for women, as the reference), because gender was not captured by Add Health.

Additional factors, however, may impact whether people *report* their non-hetero- sexual or romantic attractions—particularly, their intersecting experiences of racism, homophobia, sexism, and cultural expression or repression (Adan Sanchez, 2017; Aranda et al., 2015; Balsam et al., 2011). These oppressions also powerfully determine which people are stopped by police. In later models we therefore include race/ethnicity (Asian/Pacific Islander, Black, Hispanic, Native American, White, or Other Race/Ethnicity) and parental nativity (whether one's identified father or mother was born in vs. outside of the US) as covariates (Adan Sanchez, 2017; Mittleman, 2018). In contrast, we do not adjust for criminalized behaviors given that many of these activities result from structural stigma experienced by SM youth and do not cause SM status, nor are they likely to influence SM status reporting.

To focus on the gendered processes that we think give rise to any differences we observe by binary sex, we refer to female people and male people as “women” and “men,” respectively, throughout the paper that follows. This is to avoid suggesting that we think it is essential sex differences *per se* that drive different disparities between people of different binary sexes.

### 2.5 Analysis

Of the 15,197 respondents who participated in Wave 3 of Add Health, 14,987 (98.6%) had complete data on all exposures and covariates; 14,907 (98.1%) also had data on at least one of our three outcomes, with missingness rates for any one variable not exceeding 1.1%. We thus pursue a complete case analysis, as such low rates of missingness are unlikely to generate meaningful bias. We do, however, (A) apply weights that account both for Add Health's sampling design and study attrition between Wave 1 and Wave 3 and (B) adjust our standard errors accordingly using a first-order Taylor linearization (“robust” variance estimation).

On these weighted data we fit three main models. Model 1 only includes terms for sexual minority status, age, and sex. Model 2 introduces a sexual minority status by binary sex interaction, reflecting the gendered nature of policing risk and of homophobia. Model 3 adds parental nativity and respondent race/ethnicity as covariates. We fit these for each of our outcomes (3) and each of our definitions of sexual minority status (4). We fit logistic regression models for whether respondents had ever been stopped by the police, Poisson regression models for the number of times respondents reported ever being stopped, and accelerated failure time models assuming a Weibull distribution for age at first stop. Once exponentiated, estimates from these models represent the multiplicative increase (or decrease) in the outcome comparing sexual minorities to their heterosexual peers: their odds of ever being stopped, their number of stops to date, and their “survival time,” which here means the number of years respondents lived before they were first stopped by the police.

In sensitivity analyses, we re-fit our models for whether respondents had ever been stopped using a log-binomial approach, which calculate risk ratios as opposed to odds ratios (but can have trouble converging, particularly in cases where there are small cell sizes) (Williamson et al., 2013).

### 2.6 Secondary analyses

Given how powerfully race and racism shape policing in the US (McLeod et al., 2020; Weitzer, 1996; The President’ s Commission on Law Enforcement and Administration of Justice, 1967; National Advisory Commission on Civil Disorders, 1968), we fit Model 3 again in race/ethnicity-specific, stratified samples. These models are underpowered and unstable given very small race-sex-SM status cells (Appendix 3), especially for more restrictive SM definitions, but serve as an informative exploratory analysis for future researchers in this area.

## 3 Results

Table 1 presents unweighted sample characteristics, overall and for sexual minority groups. The percentage of the sample coded as a sexual minority varied widely depending on our definition, from a low of 3% who identified as bisexual, “mostly homosexual,” or “100% homosexual” to a high of 13% who reported at least some sexual or romantic attraction to people of the same binary sex or who did not know how to define their sexual orientation. Roughly half of the full sample identified as male, but male people were the minority of SM people across definitions (29–40%). Race/ethnicity was relatively consistent between the full sample and our SM sample, with a somewhat lower proportion of Asian & Pacific Islander and Black people coded as SMs than the full sample and a somewhat higher proportion of Native American and White people coded as SMs. Roughly 20% of our full sample had ever been stopped by the police, a rate consistent with national statistics on annual rates of police contact at the time (Langan et al., 2001). Approximately 22–24% of SMs in our sample reported having been stopped, with all groups reporting their mean age at first stop being about 21. Some children in our sample, however, reported experiencing their first stop as young as 10.

**Table 1:** Weighted sample characteristics, overall and for sexual minority groups.

Variable	Full Sample	SM Definition*			
		1	2	3	4
*Sexual Minority Status*					
Definition 1	3%				
Definition 2	9%				
Definition 3	12%				
Definition 4	12%				
Asexual	<1%				
Age	21.82 (1.86)	21.7 (1.78)	21.7 (1.82)	21.72 (1.82)	21.75 (1.85)
Sex (Male)	51%	40%	29%	31%	32%
*Race/Ethnicity*					
API	4%	2%	3%	4%	4%
Black	16%	10%	12%	11%	12%
Hispanic	12%	13%	11%	11%	11%
Native American	3%	4%	4%	4%	4%
White	66%	71%	71%	70%	70%
Parent born outside the US	16%	17%	14%	15%	16%
*Police Stop Outcomes*					
Ever stopped	21%	24%	25%	25%	24%
Number of stops	0.39 (0.88)	0.42 (0.84)	0.44 (0.88)	0.43 (0.88)	0.43 (0.87)
Age at First Stop	21.01 (2.68)	20.91 (2.65)	20.72 (2.78)	20.76 (2.75)	20.8 (2.78)

*Complete case n (range, depending on outcome)*	*14883–14894*	*464–467*	*1405–1406*	*1849–1850*	*1867–1869*

Note: Statistics presented are means/prevalences, with SDs included in parentheses for continuous variables. All statistics were weighted to make them representative of Add Health's original target population (US adolescents at baseline, during the 1994–1995 school year). Our four definitions of SM status, assessed in Wave 3, are: 1) Identified as lesbian, gay, or bisexual, i.e. responded “bisexual,” “mostly homosexual,” or “100% homosexual”; 2) additionally including those who reported ever being romantically attracted to someone of their same binary sex; 3) additionally including those who responded “mostly heterosexual (straight), but somewhat attracted to people of [their] own sex”; and 4) additionally including those who reported that they did not know how to describe their sexual orientation. Less than 1% were classified as asexual. Since heterosexuality is also redefined as our definition of SM changes, no single column for heterosexual respondents could be included.

**Fig. 1:**
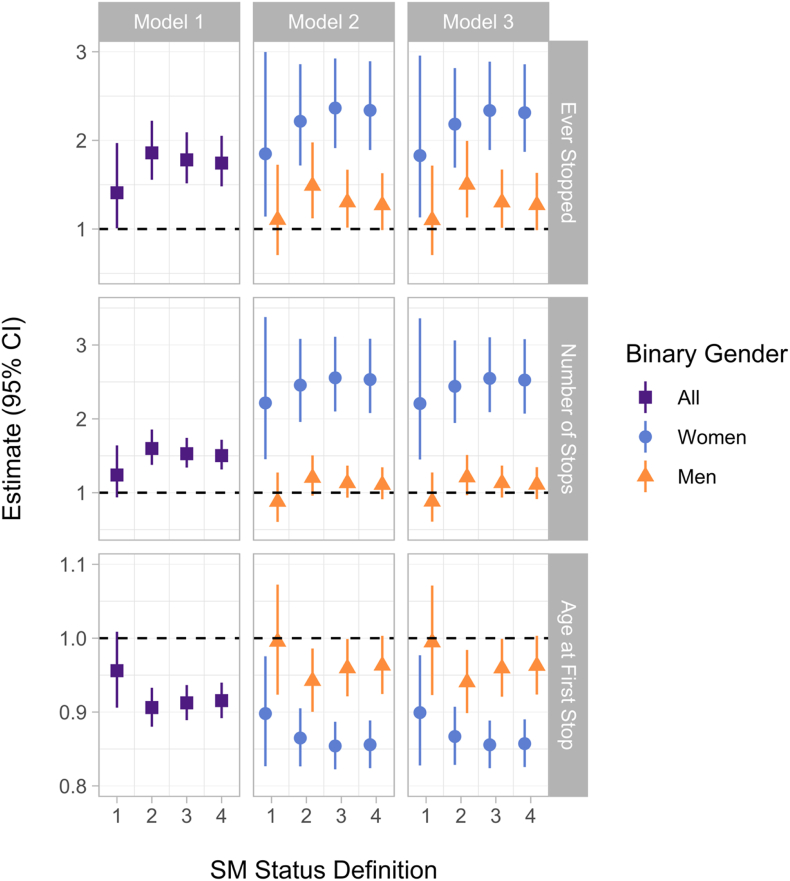
Estimated SM disparities in policing outcomes, by model, SM definition, and sex. Note: Results for different SM status definitions and outcomes are estimated using separate models run on Add Health data from its Wave 3 survey. Estimates for chances of ever being stopped represent odds ratios (logistic models); for number of stops, count ratios (Poisson models); and for age when each participant was first stopped, survival ratios(accelerated failure time models).

Fig. 1 displays results from our outcome models, with estimates plotted separately for all respondents, women, and men (men's estimates being a linear combination of the main effect for SM status and an interaction between gender [proxied by sex] and SM status). For a tabular form of these estimates (including p-values), see Appendix Table 2.

Overall—across both binary sexes, as estimated by Model 1—SMs had higher odds of ever being stopped, were stopped a greater number of times, and were stopped at younger ages relative to their heterosexual peers. These results, however, were weaker and confidence intervals included the null when sexual minority status was defined only as being bisexual, “mostly homosexual,” or “100% homosexual” (Definition 1). Estimated inequities were both larger and more efficiently estimated when our sexual minority definitions were expanded. For example, when we also included people who had ever been romantically attracted to someone of the same sex (Definition 2), SM inequities in ever being stopped by the police shifted from a Definition 1 OR of 1.41 (95% CI = 1.01–1.97, p = 0.045) to 1.86 (CI = 1.56–2.22, p < 0.001); for number of stops, from a count ratio of 1.23 (CI = 0.94, 1.64; p = 0.132) to 1.60 (CI = 1.38, 1.86; p < 0.001); and for age at first stop, from a survival ratio of 0.96 (CI = 0.91–1.01, p = 0.100) to 0.91 (CI = 0.88–0.93, p < 0.001).

Also apparent in results for Model 1 are stark differences between the police stop experiences of women and men (Appendix Table 3). Depending on our SM definition, the main effect for gender indicated that heterosexual men had between 3.71 (CI = 3.25–4.23, p < 0.001) and 3.96 (CI = 3.49–4.49, p < 0.001) the odds of ever being stopped, were stopped 3.97 (CI = 3.51–4.48, p < 0.001) to 4.15 (CI = 3.71–4.65, p < 0.001) times as often, and were stopped at younger ages (survival ratio≈0.79 in all cases, CI = 0.76–0.81, p < 0.001) compared to heterosexual women.

Similarly, SM inequity estimates differed substantially between women and men (Models 2 & 3), as reflected by large, statistically significant interaction terms between gender and SM status for nearly all models. SM inequities for ever being stopped, number of stops, and age at first stop for women were severe and statistically significant for every model and definition of SM status. Again taking Definition 2 and our model with the largest numbers of covariate adjustments (Model 3), SM women had 2.18 times higher odds of ever being stopped (CI = 1.90–2.90, p < 0.001), were stopped 2.44 times as often (CI = 1.94–3.06, p < 0.001), and were stopped at younger ages (survival ratio = 0.87, CI = 0.83–0.91, p < 0.001) than heterosexual women. In contrast, SM inequities among men were smaller, limited to whether they had ever been stopped (again taking Definition 2: OR = 1.50; CI = 1.13–1.99, p = 0.005) and their age at first stop (survival ratio = 0.94, CI = 0.90–0.98, p = 0.008), and were only discernible when we adopted more expansive definitions of SM status. Still, point estimates for SM inequities among men were non-negligible (Fig. 1). Results from sensitivity analyses refitting our models for whether participants had ever been stopped using log-binomial models yielded similar results, though risk ratios were (mechanically) somewhat attenuated relative to our odds ratios (Appendix Table 4).

**Fig. 2:**
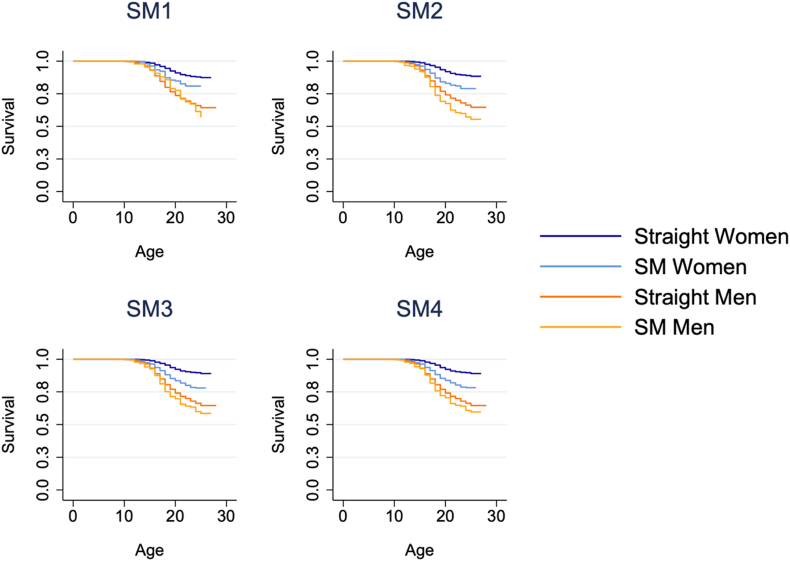
Kaplan-Meier survival curves of time until first being stopped by the police, by sexual minority status and binary gender Note: SM1, SM2, etc. are abbreviations of “sexual minority definition 1,” “sexual minority definition 2,” and so on. Each panel is derived from a separate model.

**Fig. 3:**
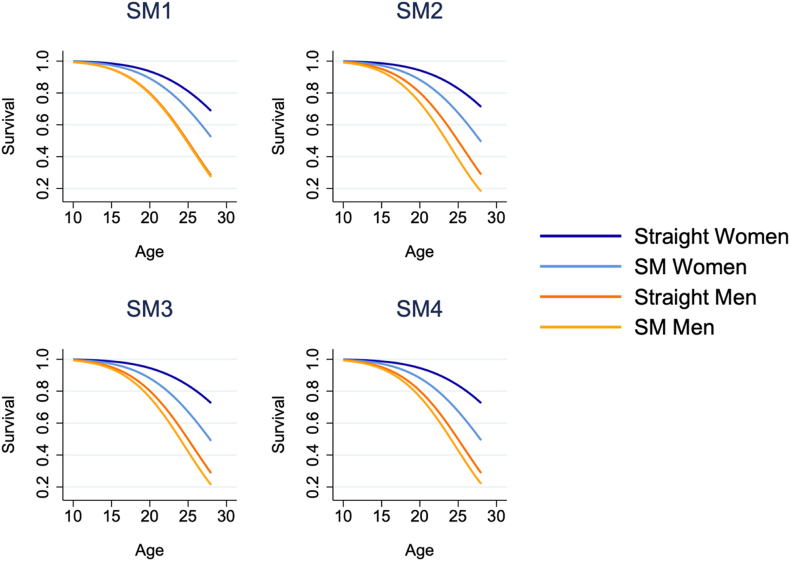
Estimated survival curves of time until first being stopped by the police from accelerated failure time models, by sexual minority status and binary gender Note: SM1, SM2, etc. are abbreviations of “sexual minority definition 1,” “sexual minority definition 2,” and so on. Each panel is derived from a separate model.

Survival times are better visualized in Fig. 2, Fig. 3. In Fig. 2, we present unadjusted Kaplan-Meier curves, with the underlying data again weighted to make the Add Health Wave 3 sample nationally representative. In Fig. 3, we present predicted survival curves according to our accelerated failure time estimates from Model 3, taking all covariates at their means. Both figures make it clear that (A) men were more likely to be stopped by the police by their young adulthood and were stopped at younger ages; (B) inequities are larger for SM vs. heterosexual women than for men, and SM inequities among men only emerge once more expansive definitions of SM status are adopted; and (C) SM men are least likely to survive into young adulthood without being stopped by the police. Following age, race/ethnicity, and parental nativity adjustment, our failure time models predict that roughly 75% of SM men were stopped by their late 20s compared to roughly 67% of heterosexual men; meanwhile, roughly half of SM women were stopped by their late 20s compared to roughly 25% of heterosexual women.

### 3.1 Secondary analyses

Power in stratified and race/ethnicity-SM interaction models was insufficient to make firm conclusions about whether SM inequities differed across race/ethnicity. Very small cell sizes (Appendix 5) meant that point estimates within the same racial/ethnic groups sometimes switched direction across SM definitions, and the ranking of point estimates across race/ethnicity also switched back and forth across SM definitions and outcomes (Appendix 6). Still, some race/ethnicity-gender-specific SM inequity estimates were extremely large, suggesting that investigating this in larger samples could reveal even more severe inequities for specific race/ethnicity-gender subgroups.

## 4 Discussion

Our findings—the first to examine disparities in police contact by SM status at a population level—show sexual minority youth were more likely to be stopped, were stopped a greater number of times (at least among SM women), and were stopped at younger ages than their heterosexual counterparts. SM women experienced particularly large inequities relative to heterosexual women, while SM men were more likely to be stopped than any other group.

This has troubling implications for SM health and health inequities. More police contact could undermine mental health, drive adoption of unhealthy coping behaviors, and worsen physical health for SMs (Everson-Rose et al., 2015; Geller et al., 2014; Hatzenbuehler et al., 2010; Jackson et al., 2019; Jahn et al., 2021; Sewell & Jefferson, 2016). Our data identifies contact that occurs at formative life course stages for socioemotional and physical development (Choudhury et al., 2006; Fuhrmann et al., 2015; Romeo, 2013). Given that SMs experience poorer health potentially unrelated to police contact, these adverse outcomes may snowball: engaging in riskier sexual activity as a part of coping with stress and poor mental health, for example, would be especially high risk in the context of disproportionately high rates of teen pregnancy among sexual minority girls (Charlton et al., 2018) and higher rates of sexually transmitted infections among sexual minority youth writ large (Everett, 2013). In other words, even if the association between police contact and health were comparable across sexual orientation, the inequitable exposure to police stops we observe among SM youth suggests that police contact may *exacerbate* existing health inequities across sexual minority status.

Sexual orientation-based inequities in policing could be caused by several, likely intersecting forces: socioeconomic marginalization, place-based targeting, or interpersonal police discrimination. First, LGBTQ people experience harsh socioeconomic marginalization: high rates of youth and adult homelessness (Morton et al., 2017; Wilson et al., 2020), accompanied by housing discrimination (Levy et al., 2017; Mallory & Sears, 2016); widespread employment discrimination (Sears et al., 2021); pay disparities that are especially severe for queer women and trans people of all genders (The HRC Foundation, 2022) etc. Poverty and homelessness likely then put LGBTQ people at higher risk of police contact, given the criminalization of homelessness (Dholakia and Jersey, 2022; Herring et al., 2019; The National Law Center on Homelessness & Poverty and The National Coalition for the Homeless, 2009), extralegal survival work (e.g., sex work (North and Vox, 2019)), and poverty itself (Collins, 2007; Edelman, 2017; Harvard Criminal Justice Policy Program and Human Rights Watch, 2017). Meanwhile, daily and life course experiences of discrimination, as well as structural homophobia in the form of second-class legal status under state and federal law, have been shown to drive “minority stress,” poor mental health, and adoption of criminalized coping behaviors, such as underage drinking or illicit drug use (Cabaj, 2000; Chien et al., 2022; English et al., 2022; Lehavot & Simoni, 2011; McCabe et al., 2010; Moody et al., 2018; Prairie et al., 2022; Raifman et al., 2017; Weber, 2008). This is compounded by alcohol and tobacco companies’ ongoing history of targeting LGBTQ people in their marketing (Elliott, 2011; Glissmeyer et al., 2018; Horgos, 2019; Spivey et al., 2018; Tufft, 2015). SM substance use inequities and their sequelae may attract police attention and further contribute to disparities in police contact. Second, LGBTQ spaces have historically been, and continue to be, targeted for disproportionate discretionary enforcement of infractions like noise complaints, public lewdness, liquor licensing, and for how LGBTQ people dress or have sex (Mallory et al., 2015). Seeking out supportive spaces built for, or primarily frequented by, fellow LGBTQ people could thus put sexual minorities at higher risk of police harassment.

Third, homophobic police officers may simply target (consciously or unconsciously) sexual minorities because of personal homophobic animus. Surveys of police officers around the time of Add Health's Wave 3 showed that 25% believed lesbianism was “unnatural” and 11% that it was “disgusting,” with much larger proportions (53% and 27%, respectively) reporting those attitudes towards gay men (Bernstein & Kostelac, 2002). Nearly half of police chiefs in Texas at the time reported that they would be uncomfortable working with a gay person, and 62% believed homosexuality constituted “moral turpitude” (grounds for being fired from a police job in several states) (Lyons et al., 2008). More recent accounts confirm a harrowing antagonism against sexual minorities even within police departments, with sexual minority officers reporting decades of bullying and death threats from their heterosexual peers (Lam, 2019). This accords with survey data from LGBTQ communities (Mallory et al., 2015): in a 2014 study surveying a national sample of LGBT people and people living with HIV, 73% had been stopped by police at some point in the last five years (Lambda Legal, 2014). A sizeable proportion of these reported hostile attitudes (21%), verbal assault (14%), sexual harassment (3%), and physical assault (2%) from police officers. Similar surveys abound: in 2013, for example, one survey found that 6% of all anti-LGBT violence in the previous year was perpetrated by police officers themselves, including 23% of all perpetrators who were not known to the victim (National Coalition of Anti-Violence Programs, 2013). The extent to which socioeconomic marginalization, place-based targeting, or interpersonal police discrimination are responsible for the inequities we show here is a question for future research.

Importantly, we show that drivers of SM policing inequities apply unequally across gender—i.e., more strongly for women. Our data cannot determine why. Any of the above mediation processes could be modified by gender: deviations from hegemonic sex and gender norms could be more severely punished for SM women than SM men in education/employment/housing or via explicit police homophobia, or discrepancies between the place-based risks of SM women and heterosexual women could be larger than those between SM men and heterosexual men. All are plausible. Socioeconomically, pay disparities for LGBTQ women are more severe than they are for men, with LGBTQ women making 13% less than cis-heterosexual women compared to a gap of 4% experienced by LGBTQ men as of 2021 (The HRC Foundation, 2022). Cisgender bisexual women compare only to trans people in their levels of poverty across gender and sexual orientation, at nearly 3 in 10, with poverty rates and pay gaps being especially severe for Black and Latina LGBTQ women—especially, trans women. Some evidence suggests girls' substance use may also be more responsive to structural oppression, e.g., in the form of anti-LGBTQ state policies (Chien et al., 2022). In terms of interpersonal homophobia from police and other authority figures, enforcement of hegemonic femininity presentation is part of the founding origins of the US criminal legal system for women: many women's prisons were explicitly created to provide “moral training” to turn deviant, particularly white, women into proper “wives, mothers, and educators of children” (Ryan, 2022) and punish Black women for supposed innate promiscuity (Gross, 2015). Such explicit racialized gender and sexuality enforcement may echo in police stop disparities today. Further, stringent femininity enforcement appears to start early in life, when the school-to-prison pipeline (Bacher-Hicks et al., 2021) can drive police contact: while sexual minority girls have been found to have 95% higher odds of experiencing punitive school discipline, no equivalent disparities have been observed among boys (Mittleman, 2018). And in terms of place-based oppression, journalists have documented severe, persistent, discretionary legal enforcement of SM women's night clubs, to the point of forcing those institutions to close (Low et al., 2020). Whether this is differential across gender is unclear.

Understanding these forces is critical for unpacking how much the policing inequities we observe here contribute to dramatic sexual orientation-based inequities in incarceration, an exposure with profound, lifelong consequences for health and aging. In 2011, despite representing less than 4% of the US population (Gates, 2011), LGBTQ women made up more than 40% of people incarcerated in US women's prisons (Meyer et al., 2017). This proportion was nearly identical to that of youth detention centers (Movement Advancement Project, 2017) and may, disturbingly, represent a historic low: in the 1960s, studies in New York City prisons put that proportion as high as 75% (Ryan, 2022). Understanding what drives SM policing inequities may be key to intervening early to prevent SM inequities in incarceration.

Importantly, sexual orientation-based policing and incarceration inequities may be a particular concern for SM youth who do not explicitly identify as lesbian, gay, or bisexual (LGB), or who are reticent to report themselves as such. SM inequities in policing were substantially larger when we adopted broader definitions of sexual minority status, suggesting that SM youth who do not explicitly identify as SMs are at especial risk. Plausibly, SM youth who identified as lesbian, gay, or bisexual may have had more social support and economic stability, reducing the interpersonal and material risk of being publicly out and also reducing their risk of police contact. Regardless, screening tools that only ask whether someone identifies as LGB are likely to miss SM youth at highest risk of being policed.

### 4.1 Implications for practice and research

Given substantially heightened police contact among SM youth, health care providers, social workers, therapists, and educators working with them should explicitly combat homophobic and criminal legal system stigma in their care and screen for police contact and its psychological sequelae. Further, professionals in these fields working with sexual minority *adults* should be conscious that their clients are much more likely to have experienced police contact in their youth, which could plausibly have been formative to their understanding of their social position, internalized homophobic stigma, and self-concept (Choudhury et al., 2006; Fuhrmann et al., 2015; Hatzenbuehler et al., 2013; Stoudt et al., 2012). Continued qualitative work on the ways police contact shapes queer youth's development—and quantitative work on the mediators and consequences of their heightened police contact—is needed.

The relationships we present here likely vary across race/ethnicity-gender groups. The imprecise and unstable results from our secondary analyses on race/ethnicity-stratified samples indicate that we will need more and better data collection efforts on LGBTQ people and their interactions with the criminal legal system. This will require either substantially larger representative cohorts (large enough to capture hundreds or thousands of sexual minority non-binary people, women, and men of each racial/ethnic group), disproportionate stratified sampling to ensure these groups are (over-)represented, or studies of specific groups (e,g., Black SM women) powered to produce efficiently estimated means that could be compared to the means for heterosexuals and other racial/ethnic groups in similar surveys launched at similar times. These studies should ask people about contact and the quality of that contact, as past research has demonstrated that youth of color differ from their White counterparts not only in the frequency of their police contact but also its invasiveness (Geller, 2021). Given the extremely high proportions of SM young people who are being stopped by police and the hugely disproportionate burden of incarceration on LGBTQ people, this is a critical priority for research on LGBTQ health, particularly the health of LGBTQ people of color (Feelemyer et al., 2021; Khan et al., 2021).

Finally, recent calls from major public health organizations underscore that criminal legal system contact, in particular police violence and incarceration, is a major challenge to the public's health—but that it does not have to be. Organizations such as the American Public Health Association have called for policymakers to adopt an abolitionist approach that obviates the need for police contact via expanded social services and universal access to basic needs such as housing, food, education, healthcare, and mental health supports—under the premise that crime is largely driven by economic necessity or psychological duress (American Public Health Association, 2018, 2020). Our results emphasize that this structural orientation to eliminating the health harms of criminal legal contact may have especially positive impacts on the health of sexual minorities.

In the meantime, independent public offices that receive and investigate complaints of police misconduct—such as New York City's Civilian Complaint Review Board—should add voluntary questions about sexual orientation to their intake forms, creating a public record of police mistreatment report disparities. (The CCRB already collects voluntary self-reports of gender and race.) Cities that lack such data-collecting civilian oversight boards should establish them. Existing programmatic solutions, such as implicit bias trainings, have so far proved to be unpromising paths forward, failing to change long-term attitudes or prevent misconduct (Forscher et al., 2019; Lai et al., 2016; Worden et al., 2020); in any case, trainings and the accountability practices of supervisors at the time these data were collected were insufficient to close sexual orientation-based inequities.

### 4.2 Strengths & limitations

Our analysis has limitations. First, we lack information on gender identity, relying instead on self-reported binary sex. This both prohibits our ability to measure inequities affecting trans and non-binary people and likely introduces measurement error into our assessments of sexual minority status and of gender oppression exposures. Second, small cell sizes for any specific racial/ethnic group within our SM sample preclude our ability to speak to heterogeneity across race/ethnicity. Third, we rely on retrospective self-report for our policing outcomes, which may introduce measurement error (especially for age at first stop). Fourth, we lack information on the nature of police stops or their consequences, particularly reports of discrimination, mistreatment, or respondents' psychological responses to stops, limiting our ability to paint a full picture of SMs’ experiences with police.

Further, our results may suffer from selection bias. SM people and those experiencing police contact may have both disproportionately dropped out of our sample. If SM teens were particularly likely to be estranged from their families, for example, the need to survive on their own might both expose them to police and increase their risk of survey attrition. This would suggest our results were underestimates. If this selection bias were more severe for particular gender or racial/ethnic groups, point estimates for specific groups may be especially underestimated. On the other hand, we include weights that account for differential attrition across a wide array of demographic and socioeconomic factors between Wave I and Wave III. Selection bias in this analysis would thus have to be strong enough to shift our estimates conditional on accounting for selection by a rich set of variables representing respondents’ social, economic, and demographic lives. Selection bias may thus be a minor concern.

More pressingly, whether the SM inequities we present here are constant over time is an open question. Policing inequities may shift across the life course (age effects) and across generations (cohort effects). The respondents in this sample are now in their late 30s and early 40s, yet more up-to-date, population-based samples of this size remain scarce. The only more recent population-level data we know of is from the Fragile Families and Child Well-Being Study (FFCWS), a national longitudinal birth cohort born in large US cities in 1998–2000 (Reichman et al., 2001). FFCWS collected information about police stops and sexuality when FFCWS children were around age 15, in 2014–2017. But these data are limited: questions about sexual orientation are vague, few in number, and identify only about 200 girls and three dozen boys as SMs when limiting to FFCWS’ nationally-representative sample. Associations between SM status and police contact in FFCWS are consistent in direction with those we find using Add Health (i.e., higher risk among SM girls; not shown), but are extremely imprecise. In sum: Add Health is the largest, most recently available, population-level information on LGBTQ policing inequities, for better (a nationally representative sample with quality sexual orientation information) and for worse (describing police contacts that occurred more than 20 years ago).

Our analysis has several additional strengths. To our knowledge, we are the first to show SM policing inequities at the national level, including stark heterogeneity across sex. We do so by analyzing police contact data about respondents' entire childhoods, followed through young adulthood, and do so for more than 15,000 youth, including as many as 1,842 SMs. Our analysis is strengthened by a flexible approach to defining SM status and careful, theoretically-motivated covariate adjustment, along with weights that help account for differential loss to follow-up. These results are particularly important as the criminalization of homosexuality resurges as a legal threat, with recent Supreme Court decisions striking down the legal justification the Supreme Court used to nullify states’ so-called “anti-sodomy” laws less than 20 years ago (Stolberg, 2022). As LGBTQ people continue to speak out against unfair treatment by the police (Green, 2020; Levin, 2019) and the specter of criminalizing LGBTQ sex rises, our estimates are an important resource for community advocacy, clinical practice, and future research to address the role of policing in LGBTQ health inequities.

## Author statement

Gabriel L. Schwartz - Data curation; Formal analysis; Methodology; Project administration; Software; Validation; Visualization; Writing - original draft; Writing - review & editing.

Jaquelyn L. Jahn - Methodology; Writing - review & editing.

Amanda Geller - Conceptualization; Data curation; Methodology; Project administration; Supervision; Writing - original draft; Writing - review & editing.

## Ethics statement

The authors declare that they have no competing interests. No specific funding supported this paper.

Add Health (the study whose public, de-identified data we analyze) is directed by Robert A. Hummer and funded by the National Institute on Aging cooperative agreements U01 AG071448 (Hummer) and U01AG071450 (Aiello and Hummer) at the University of North Carolina at Chapel Hill. Waves I-V data are from the Add Health Program Project, grant P01 HD31921 (Harris) from the Eunice Kennedy Shriver National Institute of Child Health and Human Development (NICHD), with cooperative funding from 23 other federal agencies and foundations. The views expressed in this paper do not necessarily reflect those of any of these funding agencies nor the Add Health team who collected these data.

## Declaration of competing interest

The authors declare they have no competing interests.

## Data Availability

These data are publicly available through the UNC Chapel Hill dataverse.
