# Supplementary material for: Policing sexuality: Sexual minority youth, police contact, and health inequity
Source: SSM Popul Health. 2022 Nov 17;20:101292. doi: 10.1016/j.ssmph.2022.101292 (PMC9707003; doi:10.1016/j.ssmph.2022.101292)
Supplement: Multimedia component 1 [file mmc1.docx]

**Supplemental Material**

“*Policing sexuality: sexual minority youth, police contact, and health inequity”*

**Appendix 1. Directed acyclic graph depicting hypothesized causal relationships between our exposures, outcomes, and confounders**


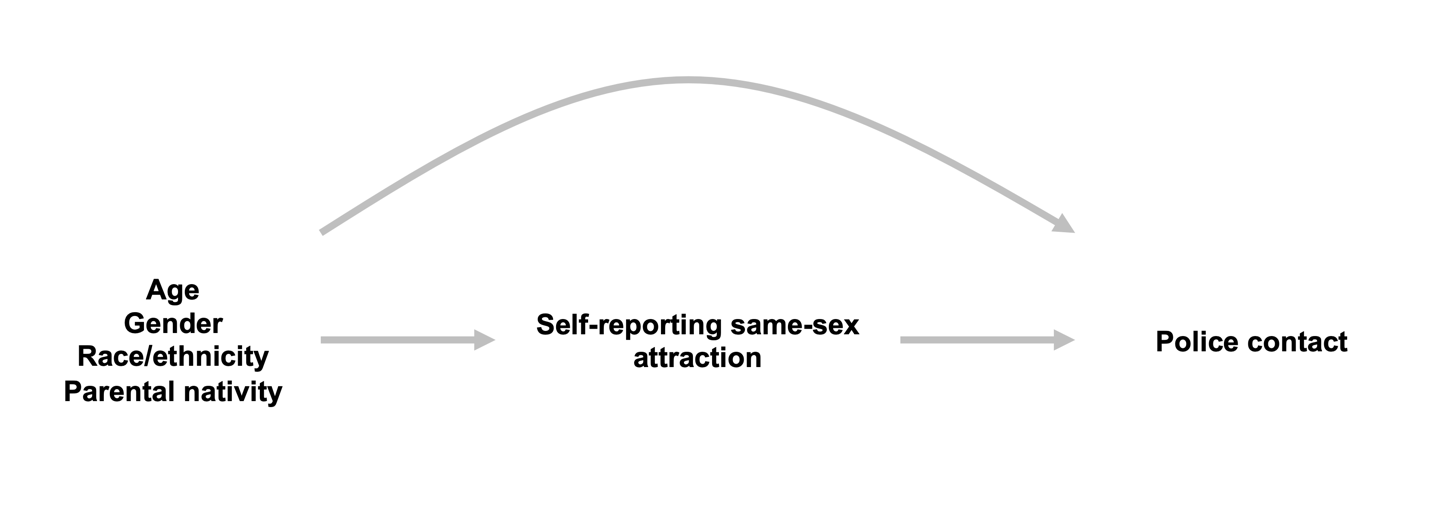


**Appendix 2. Associations between SM status and police stop outcomes, by sexual minority definition and binary sex**

| **Outcome** | **SM Definition** | **Estimate** | **Model 1** | **Model 2** | | **Model 3** | |
| --- | --- | --- | --- | --- | --- | --- | --- |
|  |  |  | *All* | *Female* | *Male* | *Female* | *Male* |
| Ever Stopped | 1 | Coef. | 1.41 | 1.85 | 1.10 | 1.83 | 1.10 |
|  |  | 95% CI | (1.01, 1.97) | (1.14, 3.00) | (0.71, 1.73) | (1.13, 2.96) | (0.71, 1.72) |
|  |  | p | 0.045 | 0.013 | 0.662 | 0.014 | 0.668 |
|  | 2 | Coef. | 1.86 | 2.22 | 1.49 | 2.18 | 1.50 |
|  |  | 95% CI | (1.56, 2.22) | (1.72, 2.86) | (1.12, 1.98) | (1.69, 2.82) | (1.13, 1.99) |
|  |  | p | < 0.001 | < 0.001 | 0.006 | < 0.001 | 0.005 |
|  | 3 | Coef. | 1.78 | 2.37 | 1.30 | 2.34 | 1.30 |
|  |  | 95% CI | (1.52, 2.09) | (1.91, 2.92) | (1.02, 1.67) | (1.89, 2.89) | (1.01, 1.67) |
|  |  | p | < 0.001 | < 0.001 | 0.038 | < 0.001 | 0.039 |
|  | 4 | Coef. | 1.74 | 2.34 | 1.27 | 2.31 | 1.27 |
|  |  | 95% CI | (1.48, 2.05) | (1.89, 2.89) | (0.99, 1.63) | (1.87, 2.86) | (0.99, 1.63) |
|  |  | p | < 0.001 | < 0.001 | 0.062 | < 0.001 | 0.062 |
| Number of Times Stopped | 1 | Coef. | 1.24 | 2.22 | 0.88 | 2.21 | 0.88 |
|  |  | 95% CI | (0.94, 1.64) | (1.45, 3.38) | (0.61, 1.27) | (1.45, 3.36) | (0.61, 1.27) |
|  |  | p | 0.132 | < 0.001 | 0.490 | < 0.001 | 0.497 |
|  | 2 | Coef. | 1.60 | 2.46 | 1.20 | 2.44 | 1.21 |
|  |  | 95% CI | (1.38, 1.86) | (1.96, 3.08) | (0.96, 1.51) | (1.94, 3.06) | (0.97, 1.51) |
|  |  | p | < 0.001 | < 0.001 | 0.108 | < 0.001 | 0.097 |
|  | 3 | Coef. | 1.53 | 2.56 | 1.13 | 2.55 | 1.13 |
|  |  | 95% CI | (1.34, 1.74) | (2.1, 3.11) | (0.93, 1.37) | (2.09, 3.10) | (0.93, 1.37) |
|  |  | p | < 0.001 | < 0.001 | 0.210 | < 0.001 | 0.205 |
|  | 4 | Coef. | 1.50 | 2.53 | 1.11 | 2.53 | 1.11 |
|  |  | 95% CI | (1.31, 1.72) | (2.08, 3.08) | (0.91, 1.34) | (2.07, 3.08) | (0.91, 1.35) |
|  |  | p | < 0.001 | < 0.001 | 0.301 | < 0.001 | 0.291 |
| Age at First Stop | 1 | Coef. | 0.96 | 0.90 | 1.00 | 0.90 | 0.99 |
|  |  | 95% CI | (0.91, 1.01) | (0.83, 0.98) | (0.92, 1.07) | (0.83, 0.98) | (0.92, 1.07) |
|  |  | p | 0.100 | 0.011 | 0.900 | 0.012 | 0.882 |
|  | 2 | Coef. | 0.91 | 0.86 | 0.94 | 0.87 | 0.94 |
|  |  | 95% CI | (0.88, 0.93) | (0.83, 0.91) | (0.90, 0.99) | (0.83, 0.91) | (0.90, 0.98) |
|  |  | p | < 0.001 | < 0.001 | 0.011 | < 0.001 | 0.008 |
|  | 3 | Coef. | 0.91 | 0.85 | 0.96 | 0.86 | 0.96 |
|  |  | 95% CI | (0.89, 0.94) | (0.82, 0.89) | (0.92, 1.00) | (0.82, 0.89) | (0.92, 1.00) |
|  |  | p | < 0.001 | < 0.001 | 0.044 | < 0.001 | 0.046 |
|  | 4 | Coef. | 0.92 | 0.86 | 0.96 | 0.86 | 0.96 |
|  |  | 95% CI | (0.89, 0.94) | (0.82, 0.89) | (0.92, 1.00) | (0.83, 0.89) | (0.92, 1.00) |
|  |  | p | < 0.001 | < 0.001 | 0.070 | < 0.001 | 0.070 |

**Appendix 3. Associations between binary gender and police stop outcomes from Model 1, by sexual minority definition (reference = women, contrast = men)**

| **SM Definition** | **Estimate** | **Ever stopped (OR)** | **Number of Times Stopped (Count Ratio)** | **Age at First Stop (Survival Ratio)** |
| --- | --- | --- | --- | --- |
| 1 | Coef. | 3.71*** | 3.97*** | 0.79*** |
|  | 95% CI | (3.25 - 4.23) | (3.51 - 4.48) | (0.77 - 0.82) |
|  | p | < 0.001 | < 0.001 | < 0.001 |
| 2 | Coef. | 3.94*** | 4.14*** | 0.79*** |
|  | 95% CI | (3.47 - 4.47) | (3.68 - 4.65) | (0.76 - 0.81) |
|  | p | < 0.001 | < 0.001 | < 0.001 |
| 3 | Coef. | 3.96*** | 4.15*** | 0.79*** |
|  | 95% CI | (3.49 - 4.49) | (3.71 - 4.65) | (0.76 - 0.81) |
|  | p | < 0.001 | < 0.001 | < 0.001 |
| 4 | Coef. | 3.95*** | 4.14*** | 0.79*** |
|  | 95% CI | (3.48 - 4.48) | (3.70 - 4.64) | (0.76 - 0.81) |
|  | p | < 0.001 | < 0.001 | < 0.001 |

**Appendix 4. Associations between SM status and whether participants were ever stopped by the police using log-binomial models (directly calculating risk ratios), by sexual minority definition and binary sex**

| **SM Definition** | **Estimate** | **Model 1** | **Model 2** | | **Model 3** | |
| --- | --- | --- | --- | --- | --- | --- |
|  |  | *All* | *Female* | *Male* | *Female* | *Male* |
| 1 | Risk Ratio | 1.24 | 1.70 | 1.07 | 1.69 | 1.08 |
|  | 95% CI | (1.00, 1.56) | (1.14, 2.53) | (0.80, 1.45) | (1.14, 2.52) | (0.80, 1.47) |
|  | p | 0.054 | 0.009 | 0.639 | 0.01 | 0.598 |
| 2 | Risk Ratio | 1.51 | 1.98 | 1.30 | 1.96 | 1.31 |
|  | 95% CI | (1.35, 1.69) | (1.61, 2.43) | (1.10, 1.54) | (1.59, 2.41) | (1.10, 1.56) |
|  | p | < 0.001 | < 0.001 | 0.003 | < 0.001 | 0.002 |
| 3 | Risk Ratio | 1.45 | 2.09 | 1.19 | 2.08 | 1.20 |
|  | 95% CI | (1.30, 1.61) | (1.76, 2.49) | (1.02, 1.40) | (1.75, 2.47) | (1.02, 1.40) |
|  | p | < 0.001 | < 0.001 | 0.029 | < 0.001 | 0.029 |
| 4 | Risk Ratio | 1.43 | 2.08 | 1.17 | 2.06 | 1.18 |
|  | 95% CI | (1.28, 1.59) | (1.74, 2.47) | (1.00, 1.38) | (1.73, 2.45) | (1.00, 1.39) |
|  | p | < 0.001 | < 0.001 | 0.05 | < 0.001 | 0.049 |

**Appendix 5. Cell sizes by sexual minority status, sexual minority status definition, binary sex, and race/ethnicity**

| **Sexual Orientation** | **Binary Sex** | **SM Definition** | **Race/Ethnicity** | | | | |
| --- | --- | --- | --- | --- | --- | --- | --- |
|  |  |  | *API* | *Black* | *Hispanic* | *Native American* | *White* |
| Sexual Minority | All | 1 | 24 | 80 | 98 | 18 | 248 |
|  |  | 2 | 75 | 272 | 225 | 55 | 785 |
|  |  | 3 | 117 | 335 | 295 | 75 | 1035 |
|  |  | 4 | 118 | 343 | 296 | 77 | 1042 |
|  | Female | 1 | 6 | 48 | 56 | 13 | 159 |
|  |  | 2 | 41 | 176 | 148 | 45 | 598 |
|  |  | 3 | 73 | 220 | 193 | 56 | 765 |
|  |  | 4 | 74 | 225 | 193 | 56 | 768 |
|  | Male | 1 | 18 | 32 | 42 | 5 | 89 |
|  |  | 2 | 34 | 96 | 77 | 10 | 187 |
|  |  | 3 | 44 | 115 | 102 | 19 | 270 |
|  |  | 4 | 44 | 118 | 103 | 21 | 274 |
| Heterosexual | All | 1 | 1100 | 3071 | 2318 | 411 | 7539 |
|  |  | 2 | 1050 | 2880 | 2191 | 375 | 7007 |
|  |  | 3 | 1008 | 2817 | 2121 | 355 | 6757 |
|  |  | 4 | 1008 | 2817 | 2121 | 355 | 6757 |
|  | Female | 1 | 537 | 1725 | 1163 | 204 | 3968 |
|  |  | 2 | 503 | 1598 | 1071 | 172 | 3533 |
|  |  | 3 | 471 | 1554 | 1026 | 161 | 3366 |
|  |  | 4 | 471 | 1554 | 1026 | 161 | 3366 |
|  | Male | 1 | 563 | 1346 | 1155 | 207 | 3571 |
|  |  | 2 | 547 | 1282 | 1120 | 203 | 3474 |
|  |  | 3 | 537 | 1263 | 1095 | 194 | 3391 |
|  |  | 4 | 537 | 1263 | 1095 | 194 | 3391 |

**Appendix 6. Associations between SM status and police stop outcomes from fitting Model 3 in race/ethnicity-specific stratified samples, by sexual minority definition**

| **Outcome** | **SM Definition** | **Women** | | | | | **Men** | | | | |
| --- | --- | --- | --- | --- | --- | --- | --- | --- | --- | --- | --- |
|  |  | *API* | *Black* | *Hispanic* | *Native American* | *White* | *API* | *Black* | *Hispanic* | *Native American* | *White* |
| Ever Stopped | 1 | 7.48 | 3.68 | 1.03 | 8.30 | 1.56 | 1.30 | 0.47 | 1.21 | 3.54 | 1.13 |
|  |  | (0.43, 130.61) | (1.18, 11.47) | (0.35, 3.06) | (1.60, 43.13) | (0.86, 2.83) | (0.21, 7.93) | (0.09, 2.49) | (0.47, 3.08) | (0.47, 26.89) | (0.65, 1.98) |
|  | 2 | 8.27 | 4.35 | 0.92 | 5.94 | 1.93 | 1.06 | 0.45 | 0.85 | 1.99 | 0.89 |
|  |  | (1.87, 36.56) | (1.69, 11.23) | (0.36, 2.30) | (2.58, 13.68) | (1.12, 3.34) | (0.26, 4.24) | (0.10, 2.05) | (0.49, 1.48) | (0.86, 4.59) | (0.54, 1.47) |
|  | 3 | 0.74 | 0.77 | 1.00 | 0.72 | 0.93 | 0.98 | 1.15 | 0.97 | 0.85 | 1.00 |
|  |  | (0.49, 1.11) | (0.61, 0.96) | (0.80, 1.24) | (0.58, 0.90) | (0.84, 1.03) | (0.79, 1.22) | (0.83, 1.60) | (0.83, 1.13) | (0.68, 1.06) | (0.91, 1.09) |
|  | 4 | 14.75 | 2.72 | 2.75 | 1.42 | 1.93 | 1.67 | 0.81 | 0.94 | 2.37 | 1.87 |
|  |  | (4.41, 49.33) | (1.14, 6.49) | (1.38, 5.46) | (0.45, 4.51) | (1.46, 2.56) | (0.48, 5.79) | (0.35, 1.88) | (0.41, 2.16) | (0.39, 14.30) | (1.34, 2.59) |
| Number of Times Stopped | 1 | 5.86 | 3.25 | 2.49 | 1.72 | 2.25 | 1.11 | 0.90 | 0.79 | 1.84 | 1.37 |
|  |  | (3.12, 11.01) | (1.45, 7.29) | (1.32, 4.71) | (0.65, 4.54) | (1.74, 2.91) | (0.45, 2.74) | (0.41, 1.98) | (0.44, 1.41) | (0.81, 4.19) | (1.07, 1.77) |
|  | 2 | 0.71 | 0.81 | 0.81 | 0.93 | 0.89 | 0.93 | 1.04 | 1.01 | 0.88 | 0.91 |
|  |  | (0.62, 0.80) | (0.68, 0.97) | (0.71, 0.94) | (0.76, 1.14) | (0.85, 0.94) | (0.79, 1.09) | (0.88, 1.24) | (0.87, 1.17) | (0.70, 1.10) | (0.87, 0.96) |
|  | 3 | 19.59 | 2.28 | 2.84 | 2.36 | 1.97 | 2.49 | 0.77 | 0.65 | 0.96 | 1.63 |
|  |  | (7.13, 53.83) | (1.01, 5.11) | (1.44, 5.62) | (0.85, 6.55) | (1.54, 2.54) | (0.73, 8.52) | (0.39, 1.52) | (0.30, 1.39) | (0.26, 3.60) | (1.23, 2.16) |
|  | 4 | 9.46 | 2.68 | 2.86 | 2.22 | 2.23 | 1.23 | 0.81 | 0.60 | 0.90 | 1.36 |
|  |  | (5.18, 17.26) | (1.23, 5.87) | (1.50, 5.46) | (0.97, 5.08) | (1.78, 2.80) | (0.54, 2.79) | (0.41, 1.60) | (0.33, 1.07) | (0.35, 2.28) | (1.1, 1.67) |
| Age of First Stop | 1 | 0.67 | 0.84 | 0.81 | 0.85 | 0.89 | 0.88 | 1.06 | 1.08 | 1.02 | 0.93 |
|  |  | (0.59, 0.76) | (0.71, 0.99) | (0.70, 0.93) | (0.71, 1.03) | (0.85, 0.93) | (0.76, 1.03) | (0.92, 1.21) | (0.94, 1.25) | (0.83, 1.24) | (0.89, 0.97) |
|  | 2 | 18.24 | 2.14 | 2.85 | 2.36 | 1.97 | 2.51 | 0.72 | 0.72 | 0.92 | 1.59 |
|  |  | (6.79, 48.98) | (0.95, 4.83) | (1.44, 5.63) | (0.85, 6.55) | (1.53, 2.53) | (0.73, 8.61) | (0.36, 1.44) | (0.35, 1.45) | (0.25, 3.42) | (1.2, 2.10) |
|  | 3 | 9.18 | 2.54 | 2.86 | 2.22 | 2.22 | 1.23 | 0.77 | 0.62 | 0.87 | 1.33 |
|  |  | (5.02, 16.78) | (1.15, 5.61) | (1.50, 5.46) | (0.97, 5.08) | (1.77, 2.79) | (0.54, 2.80) | (0.39, 1.55) | (0.36, 1.08) | (0.34, 2.22) | (1.08, 1.64) |
|  | 4 | 0.68 | 0.85 | 0.81 | 0.85 | 0.89 | 0.88 | 1.07 | 1.06 | 1.02 | 0.93 |
|  |  | (0.60, 0.77) | (0.72, 1.01) | (0.70, 0.93) | (0.71, 1.03) | (0.85, 0.93) | (0.76, 1.03) | (0.93, 1.23) | (0.93, 1.21) | (0.84, 1.25) | (0.89, 0.97) |
